# Understanding the influences on successful quality improvement in emergency general surgery: learning from the RCS Chole-QuIC project

**DOI:** 10.1186/s13012-019-0932-0

**Published:** 2019-08-23

**Authors:** Timothy J. Stephens, Jonathan R. Bamber, Ian J. Beckingham, Ellie Duncan, Nial F. Quiney, John F. Abercrombie, Graham Martin, On behalf of the Chole-QuIC collaborator group, Jenny Abraham, Jawad Ahmad, Ibrahim Ahmed, Melanie Andrews, Barry Appleton, Mohammad Asif, Russell Bolton, Christopher Briggs, Uchihara Bumagat, Simon Burchfield, Gemma Cochrane, Ffion Dewi, George Dovell, Sarah Dyer, Jemma Edge, Rachel Edwards, Ismay Fabre, Elizabeth Gemmill, Ewen Griffiths, Deepak Hariharan, Emma Harrington-Patel, Ahmed Hassn, Michael Hepworth, James Hewes, Sarah Hine, Marianne Hollyman, Kelly Ide, Deborah Jenner, Richard Johnson, Simon Jordan, Stavros Karamanakos, Joshua Kovoor, Neil Kukreja, Gabriele Marangoni, Nicola Metcalfe, Paul Morcous, Paul Needham, Niv Patel, Nabeel Qureshi, Niroshini Rajaretnam, Ilayaraja Rajendran, Yousuf Sabah, David L. Sanders, Andrew Sandison, Jennifer Sansom, Rashmi Seth, Vinutha Shetty, Tomas Sollei, Sean Strong, Laura Sullivan, Robert P. Sutcliffe, Lee Talbot, Gillian Taylor, Viju Varadarajan, Edward Villatoro, Judith Wardale, Simon Weaver, Tom Wiggins, Alexander Wood

**Affiliations:** 1 William Harvey Research Institute, Queen Mary University of London, c/o ACCU RESEARCH TEAM, 4th Floor, Central Tower, The Royal London Hospital, LONDON, E1 1BB United Kingdom; 2 Practicality Consulting, London, UK; 3 Department of Hepatobiliary and Pancreatic Surgery, The Queens Medical Centre, Nottingham, UK; 4 Department of Professional Standards, The Royal College of Surgeons of England, London, UK; 5 Department of Anaesthesia, Royal Surrey County Hospital, Guildford, UK; 6 The Queen’s Medical Centre, Nottingham, UK; 7 THIS Institute (The Healthcare Improvement Studies Institute), University of Cambridge, Cambridge, UK; 8 Department of Surgery, Princess of Wales Hospital, Bridgend, Wales, UK

**Keywords:** Quality improvement, Service improvement, Breakthrough collaborative, Gallstone disease, Evaluation, Normalisation process theory

## Abstract

**Background:**

Acute gallstone disease is the highest volume Emergency General Surgical presentation in the UK. Recent data indicate wide variations in the quality of care provided across the country, with national guidance for care delivery not implemented in most UK hospitals. Against this backdrop, the Royal College of Surgeons of England set up a 13-hospital quality improvement collaborative (Chole-QuIC) to support clinical teams to reduce time to surgery for patients with acute gallstone disease requiring emergency cholecystectomy.

**Methods:**

Prospective, mixed-methods process evaluation to answer the following: (1) how was the collaborative delivered by the faculty and received, understood and enacted by the participants; (2) what influenced teams’ ability to improve care for patients requiring emergency cholecystectomy? We collected and analysed a range of data including field notes, ethnographic observations of meetings, and project documentation. Analysis was based on the framework approach, informed by Normalisation Process Theory, and involved the creation of comparative case studies based on hospital performance during the project.

**Results:**

Chole-QuIC was delivered as planned and was well received and understood by participants. Four hospitals were identified as highly successful, based upon a substantial increase in the number of patients having surgery in line with national guidance. Conversely, four hospitals were identified as challenged, achieving no significant improvement. The comparative analysis indicate that six inter-related influences appeared most associated with improvement: (1) achieving clarity of purpose amongst site leads and key stakeholders; (2) capacity to lead and effective project support; (3) ideas to action; (4) learning from own and others’ experience; (5) creating additional capacity to do emergency cholecystectomies; and (6) coordinating/managing the patient pathway.

**Conclusion:**

Collaborative-based quality improvement is a viable strategy for emergency surgery but success requires the deployment of effective clinical strategies in conjunction with improvement strategies. In particular, achieving clarity of purpose about proposed changes amongst key stakeholders was a vital precursor to improvement, enabling the creation of additional surgical capacity and new pathways to be implemented effectively. Protected time, testing ideas, and the ability to learn quickly from data and experience were associated with greater impact within this cohort.

## Introduction

There is a pressing need to improve the quality and safety of peri-operative care globally [1, 2]. Reports from the UK point to a particular need for improvement in Emergency General Surgery (EGS), with concerns mounting about the quality of care provided [3–5]. One of the highest volume surgical presentations is acute gallstone disease, which in the UK counts for approximately one-third of all EGS admissions [6]. Despite this being a common condition, a recent multicentre prospective cohort study [7, 8] indicated wide variations in the quality of care provided across the country, with professional and national guidance [6, 9] not implemented in the majority of UK hospitals. Against this backdrop, the Royal College of Surgeons of England (RCS) set up a quality improvement collaborative (Chole-QuIC) to support clinical teams to reduce time to surgery for patients with acute gallstone disease requiring emergency cholecystectomy.

Improving the quality of acute healthcare services using a quality improvement (QI) collaborative approach has been attempted in a range of settings and has proved challenging, with many attempts demonstrating limited or no success [10, 11]; where success is reported, the quality of the study is often weak [12]. In brief, in a QI collaborative, clinical teams participate in a structured process to identify best practice and how to implement this, apply specific improvement methods, collect and share data and learn from the experiences of others about ways of achieving improvement [13]. QI interventions are invariably complex, with many interacting components, a large number of discretionary behaviours or actions required among those receiving the intervention and flexibility in tailoring implementation [14].

We undertook a prospective, mixed-methods process and outcome evaluation of the RCS’s Chole-QuIC project. In this paper, we present the qualitative aspects of this evaluation, combined with knowledge of the extent of sites’ improvement from our quantitative evaluation [15]. Noting both the overall positive impact of the collaborative and its variability across participating sites, we answer the following questions: (1) how was the collaborative delivered by the faculty and received, understood and enacted by the participants locally; (2) what influenced teams’ ability to improve care for patients requiring emergency cholecystectomy?

## Methods

### Summary of the Chole-QuIC project

The RCS set up the Chole-QuIC project in spring 2016. Recruitment to the collaborative was through a competitive application process; 13 of the 29 hospitals that applied were selected. Criteria for selection were willingness to commit surgical input to the programme; sufficient room for performance improvement and no concurrent/related improvement projects; surgical volume and type of hospital (e.g. teaching, tertiary referral centres) such that the cohort would represent the spectrum of hospital characteristics across the UK National Health Service (NHS).

### The Chole-QuIC intervention

The Chole-QuIC collaborative was a modified version of the IHI Breakthrough Series collaborative approach, incorporating evidence of what works for this type of QI project [16–18]. This approach was chosen because, although guidance on the optimal time to surgery for these patients exists, there was a lack of evidence on how to achieve this in practice. The goal for the collaborative was set in partnership with an expert reference group. After reviewing available guidance, “surgery within 8 days of presentation” was chosen to match current National Institute of Clinical Excellence (NICE) guidelines for acute cholecystitis (surgery within 7 days of diagnosis), incorporating an extra day from presentation for diagnosis to occur [9].

**Fig. 1:**
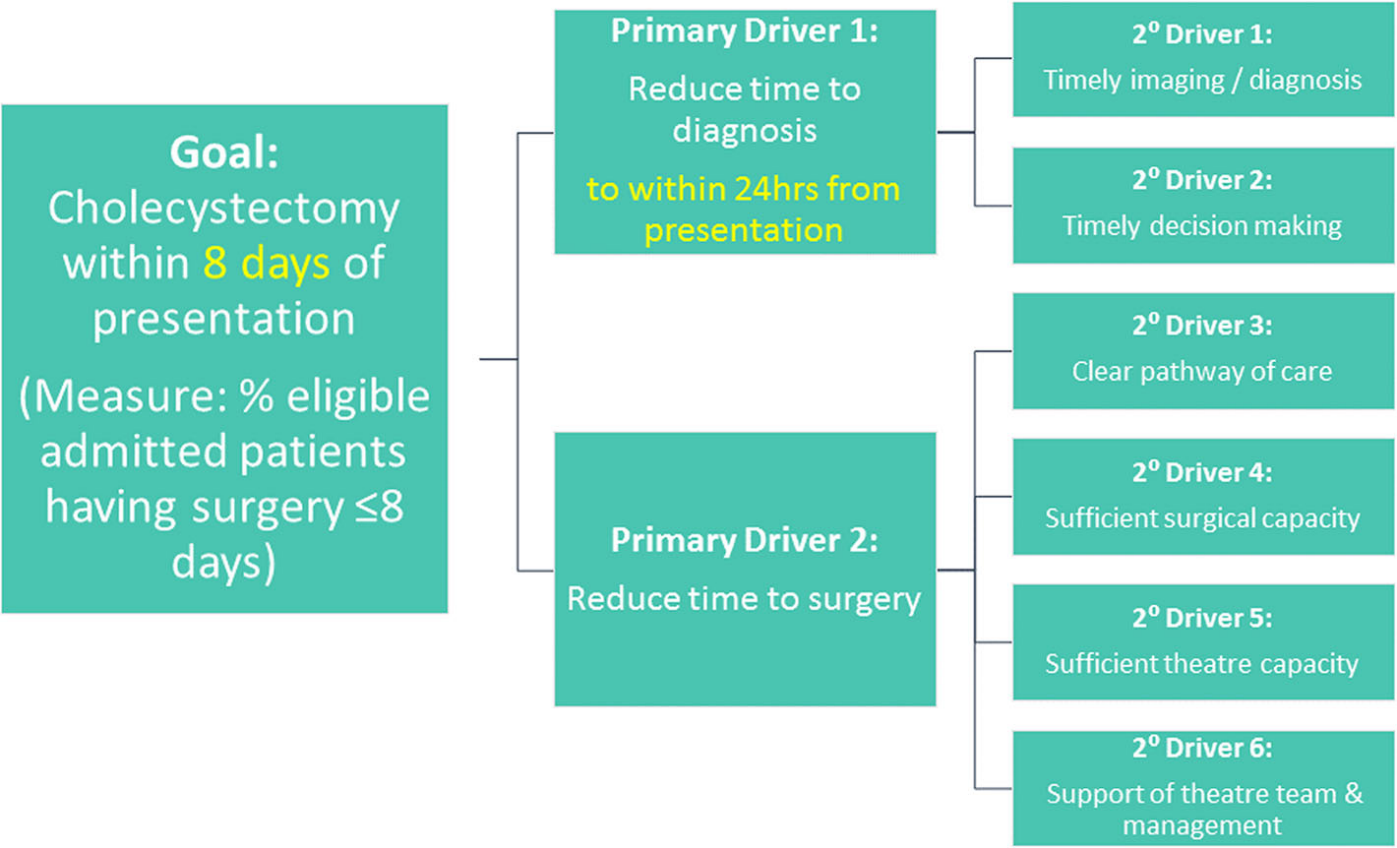
Chole-QuIC driver diagram. Chole-QuIC, Cholecystectomy Quality Improvement Collaborative

**Fig. 2:**
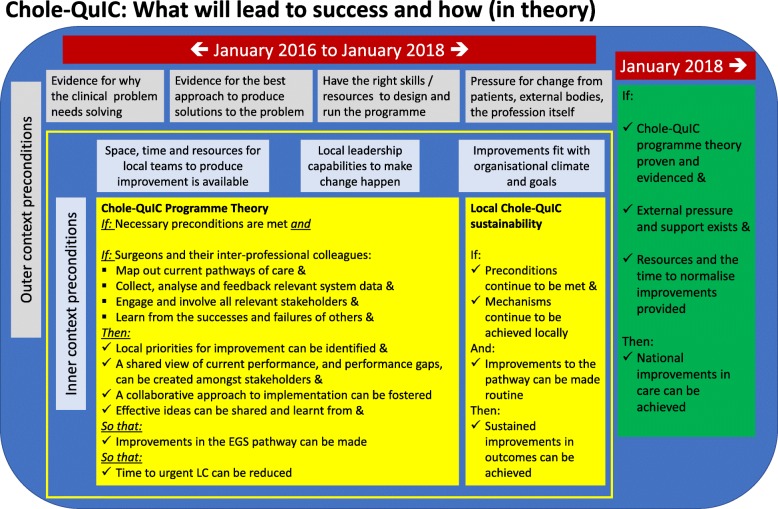
Chole-QuIC theory of change. Chole-QuIC Cholecystectomy Quality Improvement Collaborative, EGS, Emergency General Surgery/LC, laparoscopic cholecystectomy

**Fig. 3:**
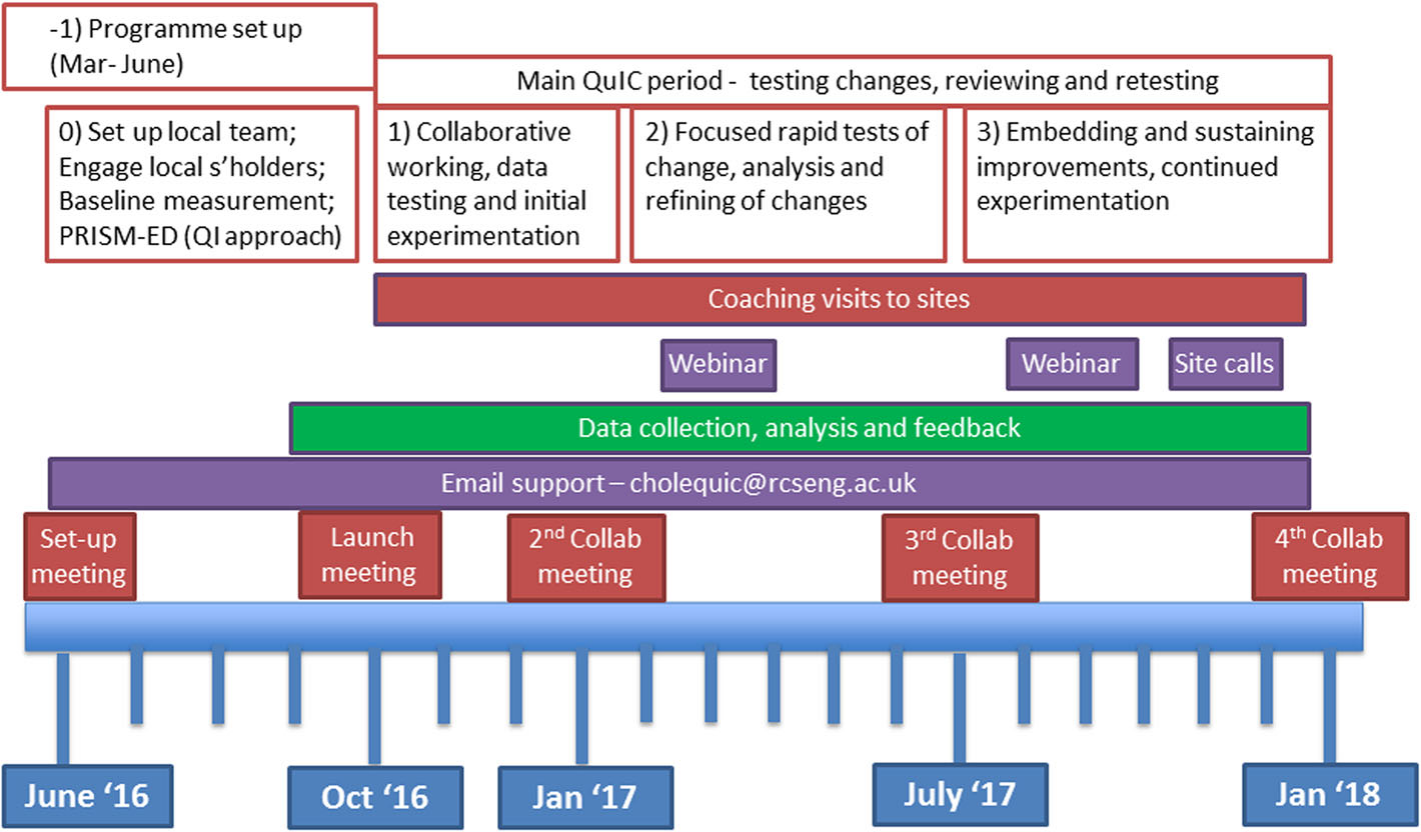
Chole-QuIC programme structure and key activities. Chole-QuIC, Cholecystectomy Quality Improvement Collaborative

We visited several hospitals that were known to be managing acute gallstone disease in line with guidance and used learning from these and from other relevant QI work (e.g. [19–22]), and, in consultation with an expert in this field of surgery (IB), developed a driver diagram (Fig. 1) and a Theory of Change (explaining the necessary conditions and the ‘how’ and ‘why’ of the intervention; Fig. 2). Improvement interventions can be considered to have a hard core, the component(s) that impacts on the main outcome of interest, and a soft periphery, the component(s) that supports getting the intervention hard core into practice [23]. The hard core of the Chole-QuIC intervention was the focus on development, testing and ultimately implementation of context-specific solutions that would move their service toward achieving the project goal. The soft periphery of the intervention were components including local process measure collection and analysis, stakeholder engagement and learning from others within the collaborative. Teams were supported through this process with a range of activities, as detailed in Fig. 3.

### Study design

We employed a partnered evaluation approach, utilising the knowledge and experience of Chole-QuIC faculty (TS, JB, NQ, IB) combined with oversight from a senior, external researcher, with expertise in the field of mixed-methods and qualitative evaluation (GM). The evaluation was approved by the ethics review committee of Queen Mary University of London [QMREC1817a]. Project findings are reported in accordance with SQUIRE guidelines [24].

### Data collection

We collected a range of data at both the collaborative-wide level (all sites) and in a purposively sampled sub-set of sites. At the collaborative level, we collated (1) field notes, compiled by project faculty (TS and JB) immediately following coaching site visits; (2) ethnographic observations, involving non-participant observation of each of the main collaborative meetings, undertaken by external researchers; 3) notes from webinars and site calls; and (4) project documentation, including slides prepared by teams and summative site reports written by the faculty for each site. We purposively sampled a sub-set of sites to take part in focus groups. We sought to achieve a maximum variation sample (in terms of surgical volume, teaching/specialist status and performance during the project). We recruited sites for focus groups at two stages (5 months into the project and at the end of the project). Focus groups ranged from 4 to 12 participants and included lead surgeons (consultant grade), surgical trainees, other junior doctors involved in the project, nursing staff, anaesthetic staff, booking co-coordinators and service managers. Ethnographic observations and focus group recordings were professionally transcribed. All sites and individuals are pseudo-anonymised.

### Data analysis

To answer question 1, we primarily used data from project documentation and from ethnographic observations of the collaborative meetings to understand participants’ response to the meetings and programme overall, using a deductive framework approach driven by our question: (a) how the programme was delivered by the faculty and (b) how it was received, understood and enacted by the participants. To answer question 2, we adopted a comparative case study approach. We identified two sets of cases: ‘highly successful hospitals’ and ‘challenged hospitals’, each containing four hospitals, using the main outcome measure for the collaborative, increase in the proportion of patients who had their surgery within 8 days of presentation [15].

**Fig. 4:**
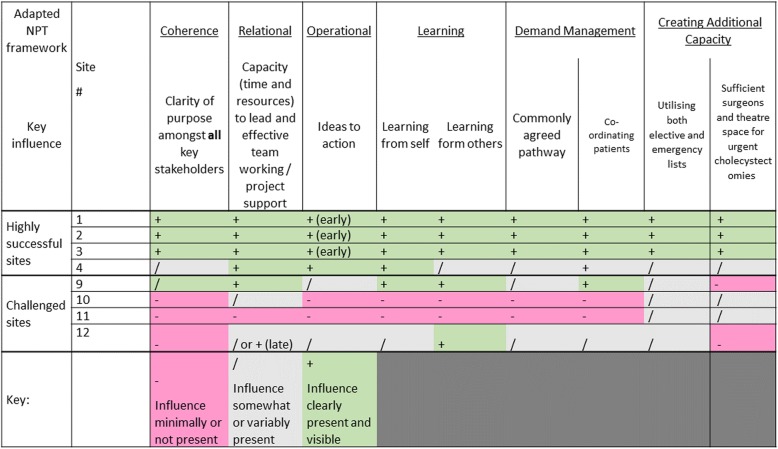
Presence or absence of main influences on successful improvement in case study sites. Chole-QuIC, Cholecystectomy Quality Improvement Collaborative. NPT normalisation process theory

Data analysis for question 2 was based on a modified form of the Framework Analysis approach [25, 26]. We generated emergent themes that seemed important to improvement success from the data for all hospitals in the cohort, sensitised by constructs from Normalisation Process Theory (NPT) [27, 28] and the Chole-QuIC Theory of Change (Fig. 2). NPT maps out the improvement process as the product of four social mechanisms (see Table 1): coherence (what individuals and teams do to make sense of a new practice); cognitive participation (what individuals and teams do to engage with new practice); collective action (what individuals and teams do to enact a new practice); and reflexive monitoring (what individuals and teams do to appraise the effects of a new practice) [27, 29]. We used the NPT as a structure to support our understanding of the social and contextual influences at play within the Chole-QuIC sites and ultimately adapted and added to the NPT constructs to reflect the themes emerging from the inductive data analysis. After identifying the subset of ‘highly successful’ and ‘challenged’ hospitals as above, we undertook a more structured deductive approach where data were further analysed using this emergent set of themes and influences. Throughout the process, manual coding of data was undertaken separately by three individuals (TS, JB, ED) and then discussed with other team members, with codes and aggregate themes agreed during regular meetings. Analysis of the case study data involved developing within-case themes, then identifying cross-case themes and patterns, looking in particular for data that might provide an understanding of what enabled or hindered successful improvement and identifying potential rival explanations. Having familiarised ourselves with the data and emergent themes, we developed a visual framework of within- and across-case patterns (Fig. 4).

**Table 1:** Description of key influences on success and related NPT construct

Description of key influences	Overall area of work	Related NPT construct
Cognitive, relational and behavioural work		
1. Achieving clarity of purpose amongst site leads and all key stakeholders	Sense-making	Coherence
2. Capacity (time and resources) to lead and effective team working/project support	Relational	Cognitive participation
3. Turing ideas into action	Making change happen	Collective action
4. Learning from own and others’ experience	Learning from change	Reflexive monitoring
Clinical process		
5. Creating additional capacity to do emergency cholecystectomies	Surgical/theatre capacity	N/A
6. Coordinating/managing the patient pathway	Patient pathway/flow	N/A

*NPT* Normalisation Process Theory

## Results

Of the 13 sites invited to join Chole-QuIC, 12 fully participated throughout the programme, attending all four collaborative meetings and 3 webinars, participating in at least 1 site visit, collecting prospective data throughout and testing improvement ideas. Site 13 withdrew voluntarily after 9 months, having engaged to only a limited extent (no attempt at service changes, incomplete data submission). In total, we collected evaluation data comprising 6 focus group transcripts (out of the 8 planned) from 5 hospitals, field notes from 17 site visits, 4 transcripts from ethnographic observations of collaborative meetings, and 12 summative site reports. We were unable to convene focus groups with two selected sites due to logistical issues, but had detailed field notes for both from site visits towards the end of the project.

### Outcome of the Chole-QuIC project

Chole-QuIC achieved its aim of demonstrating that gallstone care can be successfully improved in English and Welsh hospitals, although the extent of this improvement varied between the participating sites. Two-thirds (8/12) of participating hospitals improved care significantly, from the baseline period (April 2014 to June 2016) to the intervention period (October 2016 to December 2017) in the main outcome measure for the collaborative, proportion of patients having surgery within 8 days of presentation, even after accounting for a secular trend towards improvement among control hospitals (Table 2). Note that although intervention period results still appear low for many sites, our data from the national control group indicate the top-performing quartile of hospitals across England and Wales achieve a median of 26% (range 21–48%) of patients having their urgent laparoscopic cholecystectomy within 8 days. Four highly successful hospitals achieved a significant improvement that was well ahead of any secular trend across the rest of England and Wales, at least doubling rates of surgery compared to the baseline period. Moreover, this improvement was sustained for nine months or more during the project. Conversely, four challenged hospitals did not achieve a significant improvement against the 8-day goal in our analyses and their performance on the main outcome measure remained below the national average for the duration of the project. A full analysis of the quantitative data is published elsewhere [15].

**Table 2:** Site surgical activity and achievement of surgery within 8 days during Chole-QuIC

	Activity—all admissions for biliary disease		% Procedures within 8 days (all admissions)		Relative change from baseline		Combined model (adjusted for control group)	
	Baseline	Intervention	Baseline (%)	Intervention (%)	Relative change	95% confidence interval	Relative change	95% confidence interval
All Chole-QuIC	13,929	7944	9.4	14.6	1.56*	1.38 to 1.75	1.45*	1.29 to 1.62
Control	147,495	83,391	14.2	15.3	1.08*	1.02 to 1.14		
Site 1	521	301	8.8	25.9	2.94*	2.02 to 4.27	2.73*	1.88 to 3.96
Site 2	964	521	12.2	26.5	2.16*	1.69 to 2.77	2.01*	1.55 to 2.60
Site 3	513	355	16.8	35.2	2.10*	1.60 to 2.76	1.95*	1.47 to 2.59
Site 4	1103	629	9.9	20.8	2.09*	1.45 to 3.01	1.96*	1.50 to 2.55
Site 5	1333	770	4.6	8.6	1.88*	1.27 to 2.77	1.74*	1.22 to 2.49
Site 6	1114	619	8.5	14.7	1.72*	1.06 to 2.79	1.60*	1.19 to 2.16
Site 7	1189	627	6.7	11.2	1.68*	1.06 to 2.65	1.54*	1.11 to 2.15
Site 8	1413	900	14.4	19.6	1.35*	1.11 to 1.66	1.26*	1.01 to 1.56
Site 9	1213	684	6.5	8.3	1.28	0.88 to 1.85	1.19	0.84 to 1.68
Site 10	1476	760	8.4	8.8	1.03	0.64 to 1.66	0.97	0.72 to 1.33
Site 11	1505	793	2.9	3.0	1.02	0.59 to 1.77	0.96	0.58 to 1.59
Site 12	1585	985	16.5	14.2	0.86	0.69 to 1.09	0.8	0.64 to 100

*Significant improvement (*P* < 0.05)

Legend: Sites 1–4, highly successful group, sites 9–12, challenged group

### Delivery of the collaborative activities

The collaborative programme was delivered largely as planned, with the exception of one additional set of site calls, added to maintain teams’ momentum during autumn 2017. Site participation at each meeting was complete with 0/12 site teams missing the main meeting (and site 13 attended all meetings prior to withdrawing from the collaborative). The median size of each site’s team attending was 3 members of staff (range 1 to 6). Regarding calls and webinars, 11/13 teams participated in the first webinar, 9/12 teams participated in the second webinar and 9/12 participated in the site calls. Site visits to coach teams were delivered less than planned. We planned 26 site visits over the course of the programme (2 per site) but by the end of the programme had made only 17. All sites had at least one visit and were offered a second. Reasons for declining a second visit included lack of time to host the visit, failure to find a mutually convenient data and time (between site and Chole-QuIC teams) or site leads taking the view that a second visit was not necessary.

#### How was the collaborative received, understood and enacted by participants?

Overall, the Chole-QuIC project was well received by participants, in terms of both the clinical problem to be fixed and the approach taken to improve outcomes.

“The consultant said immediately that ‘this project was absolutely the right thing to do’, he said he knew it was ‘the way forward’ … [This] seemed to be the consensus of opinion of many of the other consultants attending.” [Ethnographic notes, January 2018 meeting]

“As the room fills up, a sense of energy gradually builds. There does seem to be a buzz in the room, a sense of anticipation. I sense that people want to be here.” [Ethnographic notes, July 2017 meeting]

Several site leads reported having wanted, or having tried unsuccessfully, to improve care for this patient group for a long time, and felt that this project, particularly with the associated support from a professional body like the RCS, was what they needed to drive improvement forward.

“He said that he had been trying for many years to get urgent cholecystectomies undertaken in his hospital but with no success…He made the comment that he felt that at their hospital they had got all the necessary ingredients to make this work but that unfortunately they ‘haven’t got the oven’.” [Ethnographic notes, October 2016 meeting]

Attendees at collaborative meetings often reported feeling motivated or re-energised by attending. In line with the programme theory behind collaborative approaches [17, 18], some found they gained ideas from those who were doing well, and insights into how to overcome challenges.

“[Participants told me] it had been very useful listening to others’ ideas. They also mentioned that they do not have a pathway as such, except possibly one that is “in their head”. So they found the session about pathways particularly useful.” [Ethnographic notes, Jan 17 meeting]

This feeling was not universal, however. Some other site leads enjoyed the social aspects of the meetings but stated that they did not derive benefit from the collaborative approach.

“…and I like the meetings, it’s nice, because I know some of the other guys from the hospitals so it’s quite a sociable thing and it’s good to go and speak to people […] but I haven’t found, I haven’t had to collaborate [with other Chole-QuIC sites]; I haven’t found the need to speak to other units independently from what we’re doing” [Focus group, highly successful group]

Overall, our data suggest that the most important aspects of the collaborative, from sites leads’ point of view, appeared to be (1) meeting up with like-minded colleagues, (2) the external drive or focus that the collaborative afforded and (3) the legitimacy conferred by Chole-QuIC’s status as a Royal College of Surgeons initiative. Whilst there was some clear evidence of cross-pollination of ideas and some communication and partnering outside meetings, we found these aspects to be much more limited.

In terms of how participants enacted the Chole-QuIC programme theory (see Fig. 2) locally, our data analysis indicated that all participating sites attempted to follow the recommended steps, but with varying degrees of fidelity. For example, some sites focussed much more on using local data collection to drive improvement than others. Through comparison of the case-study hospitals, we next examine this variability, and its positive and negative consequences, in more detail.

#### How was Chole-QuIC enacted locally by teams and what influenced success?

Our analysis highlighted the extent of cognitive, relational, and behavioural work (the ‘enactment’ of the Chole-QuIC programme locally) that site leads and their teams needed to do to improve care for this patient group. This work is described under six descriptors of key influences, alongside their related area of improvement work and related NPT constructs (Table 1). This work appeared to relate directly to hospitals’ success in achieving the project goal: the four ‘highly successful’ sites achieved these tasks effectively, while the four challenged hospitals appeared to struggle with them. We describe each separately, although they are interdependent. In particular, no single set of features could be credited for success or lack thereof; rather it was the combination of their presence or absence, and the interaction between them, that appeared important.

### Clarity of purpose amongst site leads and other stakeholders

Clarity of purpose was much better established in the highly successful than the challenged sites. In some QI projects, stakeholder engagement may be ‘desirable’ rather than necessary, but here it seemed vital, as diverse stakeholders were key to creating capacity and unblocking access to theatre lists. In one highly successful site, where the clarity of purpose was palpable amongst key staff we met, the use of a patient story seemed to galvanise everyone into action.

“I talked about my patient, who waited 18 months to see me in clinic, which is not an unusual wait here in [area] and when I met her she was having an attack of biliary colic in the waiting room, so I admitted her. My clinic is on a Monday, I operated on her the following day… Bringing her along to some of my colleagues on our clinical governance day and just having her speak about her experiences has helped put Chole-QuIC right in the forefront of people’s minds.” [Focus group, highly successful group]

In the other highly successful sites, evidence from field notes indicated a shift in culture and behaviour from willingness to change ‘in principle’ towards modified processes that were endorsed by a range of stakeholders. Leads in two sites deployed a patient story along the lines above; in all four, engagement was a clear strategy of the site lead, extending well beyond emails and convening stakeholder meetings. Leads used multiple strategies including meetings, one-to-one conversations, data feedback and opportunistic moments (e.g. corridor conversations) to ‘sell the idea’. Participants from both successful and challenged sites stressed that gaining common understanding and support for the work was difficult. A key point of divergence was that clarity of purpose was absent in all challenged sites in at least one key stakeholder group, i.e. amongst surgical colleagues, senior service managers or those gate-keeping emergency theatre lists. In challenged sites, attempts were made to engage with necessary stakeholders, but the clarity of purpose visible in the highly successful sites remained absent. The challenge seemed greater in the larger centres, where the size of these organisations, and the presence of multiple surgical teams within the two specialist centres, meant that achieving coherence across the whole group of surgical stakeholders was harder. In one site, for example, the reported attitude from one key colleague in a different surgical sub-specialty team was

“We haven’t got a problem, so we don’t need to change”. [Focus group, challenged group, emphasis added]

Field notes also suggested factors that may have affected the clarity of purpose. In one site, failure to agree on how to treat these patients made progress near impossible; in another, recurrent organisational challenges, both financial and patient flow-related, meant that creating extra surgical capacity was not a priority for the responsible managers. Again, the challenge seemed greater in the larger centres,

“Despite what seems like quite a bit of progress, what comes across most in his talk is that ‘we have a lot to do to change attitudes’. Key issues seem to be that it’s difficult to get into theatre (they are competing with other cases, e.g. cancer, and if they do get slots these tend to be late when it’s not safe to operate), there’s a reluctance to prioritise emergency patients over ‘long waiter patients’.” [Ethnographic notes, July 2017 meeting]

### Capacity to lead and effective team working/project support

We identified a divergence between highly successful and challenged sites in their success in ring-fencing time for the project. In all highly successful sites, time for the project was included in the lead’s job plan; conversely, in two challenged sites, this was never achieved, and in the other two, it was achieved only later in the project. Leading any project alongside existing clinical commitments can be challenging, especially when the lead has a role in motivating and encouraging others. As a highly successful site lead put it,

*“I’m glad I put all this work in…it wouldn’t have worked otherwise, but… I’m exhausted” [Field note, highly successful group, November 2017 site-visit]*

In one challenged site, the lead applied to include dedicated time for Chole-QuIC in his job plan but this was denied; unsurprisingly, he saw this as not only a practical disadvantage but also a signal of the limited commitment of senior management to the project overall. No site lead attempted to make change happen single-handedly; all leads built teams or support groups of varying sizes and composition. A pattern was apparent, however, in how these teams worked toward project goals. In more successful sites, professionals behaved as a coordinated, interdependent team, rather than as a working group or simply colleagues working in parallel. For example,

“One thing that [site lead] was very clear about was the Friday morning meetings. He said that although they all communicated during the week about patient coordination, they tried (generally successfully he says) to meet briefly every Friday morning at the start of the day to catch-up on more of the ongoing improvement activities.” [Field note, highly successful group, November 2016 site-visit]

“At the final event they depicted their work with a picture of a rugby team in order to illustrate that they had ‘successfully managed to get a good team together and that actually we’re pleased with ourselves for managing this’.” [Ethnographic notes, January 2018]

One challenged site did have a similarly high functioning team by the end of the programme. However, it only developed later in the project, seemingly limiting progress earlier on.

“This has been a huge learning process for me along the way, to try and lead it […] so I got some nursing time from one our very senior nurses […] And, between us I thought that the two of us could then go out into the wider [site] audience, and we would be able to manage that. And I think that was probably the wrong approach. We probably should have engaged right from the beginning with a wider team, we should have had a team of four, five, six people.” [Focus group, challenged group]

### Turning ideas into action

The Chole-QuIC approach focussed on helping teams to develop (or adapt) solutions to overcome local challenges and test them to see if they would work in practice, following the iterative Model for Improvement approach [30]. In summary, this approach entails (1) using data to understand the current position, (2) defining an improvement goal; (3) developing ideas to get from current position to the goal; (4) testing ideas in mini ‘real-world’ experiments; (5) refining and further testing ideas that work and discarding ideas that do not and (6) implement most effective ideas and continue to use data to monitor [30]. A notable point of divergence between highly successful and challenged cases was the speed with which the former put ideas into action. In the challenged cases, this translation process was much slower—or absent altogether. Four months into the project, three highly successful sites were already submitting data that showed successful moves toward the collaborative goal. The fourth was actively testing out ideas. For example, in one site, the team very quickly agreed to trial the ring-fencing of elective slots for emergency cholecystectomies to examine the impact.

“Both of us said ‘look, why don’t we keep slots free on our lists’. My list is a Tuesday, [other surgical lead] is a Thursday… So I didn’t speak to anybody outside that [immediate theatre team] because my experience of NHS management is if you ask permission then you’re waiting six months for an answer. So very much do it, then seek forgiveness.” [Focus group, highly successful site]

The importance of maintaining this willingness to test and adapt beyond the initial change was also identified; in another highly successful site, not only had staff turned their initial ideas into action early on, but continued to revise and refine their new process over time.

*“*Minor process changes were introduced in response to data review and discussion at collaborative meetings, including additional training on the pathway during staff rotations [and they] utilised the ‘Whiteboard’ idea [from another site].”

“In July 2017, they made the bold decision to move from elective lists for admitted patients [first change] to using held slots on CEPOD [emergency theatres]…From November 2017 the team are looking to introduce an ‘as needed system’ of pulling an elective list when demand increases over a 2-week period…” [Field notes, highly successful site, October 2017 site-visit]

Conversely, in the challenged sites, a lack of (early) action was evident. This did not seem to reflect a lack of desire to improve care processes; rather, a combination of contextual factors and a reliance on a slower, more methodical planning approach was apparent. For example, in one site, data from field notes and meetings showed that several months were spent designing, agreeing and planning the implementation of a new pathway, with associated paperwork. Unfortunately, however, the pathway proved unsuccessful: colleagues simply did not use it. The project team found it hard to recover from this setback, particularly in the context of a time-limited project, and by the end of the project, little progress toward to the Chole-QuIC goal had been made.

### Learning from own and others’ experience of change

Learning from sites’ own data was a cornerstone of the Chole-QuIC Programme Theory. Whilst all teams collected and collated data for the project, the perceived importance of this data varied. In most highly successful sites, data was collected almost contemporaneously and then reviewed to track progress.

“Their goal has been to have 80% of patients within eight days and, they tell us, they’re heading in the right direction to achieve that. This is at least in part due to data informing how they organise the service and guiding them to focus on “gaps between goal and reality”. [Ethnographic notes, July 17 meeting]

Data used was not always in the form prescribed by the Chole-QuIC core team. Field notes indicated that in three of the four highly successful sites, a variety of data and other local intelligence was used, including using coding data and more traditional theatre logbook checks, to monitor new processes and how these were working. For the challenged sites, data collection and analysis were deprioritised, so that the information available was retrospective in nature, rather than providing timely and actionable insights into the impact of activities. In some sites, collecting data seemed to be an activity undertaken for the RCS project team rather than for teams themselves to analyse and monitor progress.

“[Site lead] said how useful it was to review their data. I asked how often they had been doing this and he, a little apologetically, replied that they had not really had the time to do this by themselves.” [Field note, Challenged group, October 2017 site-visit]

### Creation of additional capacity for emergency cholecystectomies

Besides the four activities discussed above, relating to the NPT constructs of cognitive and relational and behavioural work, we identified two further influences that distinguished highly successful from challenged sites, relating to clinical processes. First, creating additional surgical capacity was essential. Approaches varied between sites. Successful sites tended to use a dual strategy, ring-fencing elective slots for emergency work whilst simultaneously working on engaging colleagues in theatres with the concept that some emergency cholecystectomies belonged on the emergency theatre lists, even though historically these cases had been afforded much lower priority. This two-pronged attempt to create capacity, repurposing elective space plus optimising use of emergency theatres, appeared to produce the most successful results.

Conversely, in the challenged sites, difficulties in creating additional capacity were a major barrier. Here, competing clinical priorities prevented the addition of more emergency cholecystectomies onto already overburdened emergency lists and made it difficult to find suitable elective lists that could be ring-fenced or repurposed for these procedures.

Moderator: So you said that the ring-fenced capacity has helped but I think you also alluded to the fact there’s nowhere near enough capacity. So, what’s getting in the way of getting more capacity? [Everyone laughs]

Participant: Everybody in the hospital wants more capacity… [Focus group, challenged group]

### Managing and coordinating demand across an agreed pathway

Second, in terms of clinical processes, the four highly successful sites all succeeded in reaching agreement across stakeholders on the appropriate clinical pathway for this patient group. For example, in one, both a shared understanding of the pathway and an effective (albeit rather ‘low-tech’) mechanism for patient coordination were in evidence.

“The biggest success that we’ve had has been the Chole-QuIC board that [team members] came up with and it was just a board… we put anybody, any patient, on there with a putative diagnosis of acute biliary disease, whether it’s right or wrong doesn’t matter, it’s about getting them up there and then scrubbing those patients out a couple of times a week and saying ‘yes this one is, no this one isn’t’[...] as the patients get identified and targeted.” [Focus group, Highly successful group]

Conversely, in three of the four challenged sites, neither a pathway (whether formally documented or informally understood) nor mechanisms of coordination between different parts of the service were present. In one larger challenged site, recognition of the problem of coordination led the lead to build a business case for a biliary coordinator, a role that two other (successful) Chole-QuIC sites had created. However, by the end of the project, no one had been appointed and the issue of coordination remained. In another challenged site, a pathway was agreed amongst consultant surgeons, but to the lead’s frustration, it was not followed in practice.

“In fact, many of the surgeons were carrying out practice which ensured that outcome goals could not be met. One of the frustrations outlined by the project lead at the final project meeting was that some surgeons are sending people for MRI scans [out with the agreed pathway], which regularly took over a week to take place.” [Ethnographic notes, January 2018 meeting]

## Discussion

This study showed that a QI collaborative approach can be effective at reducing time to surgery for patients with acute gallstone disease, but realising the approach was complex and challenging. Our study is distinguished by the use of a robust mixed-methods design which demonstrated the overall impact of the approach compared to a contemporaneous control group [15] and highlighted the differential effectiveness of the approach across 12 participating hospitals. This enabled us to identify aspects of implementation and context associated with greater impact in four ‘highly successful’ hospitals demonstrating the most statistically significant change, and less impact in four ‘challenged’ hospitals that were least successful by this measure. Our framework, guided by Normalisation Process Theory [28], but populated through comparative analysis of data from the cases of highly successful and challenged sites, suggested six sets of influences that seemed most consequential (Table 1 and Fig. 4). Intensive work was required to ensure that all key stakeholders had a shared understanding of, and agreement with, the purpose and benefits of rapid surgical intervention; where this was in doubt, achieving improvement was more challenging. However, clarity of purpose was a necessary, but not sufficient, condition for improvement (Fig. 4). Sites also systematically diverged in their handling of practical issues, such as protected time within job plans, functional team-working, and rapidly turning ideas into action. It was a combination of these influences that characterised the highly successful sites in Chole-QuIC. Other key factors for success, more specific to emergency surgery and not so readily accounted for within the NPT-informed framework, included a multi-pronged approached to creating additional theatre capacity, and agreeing on a clear pathway with effective coordination mechanisms.

Our findings, interpreted in light of relevant theory and research, allow us to offer some transferable lessons for other practitioners. Firstly, our findings contribute to the growing body of literature with equivocal findings regarding the collaborative nature of QI collaboratives [18, 31]. Engagement in Chole-QuIC was good, with consistent attendance and involvement by between 1 and 6 staff throughout from sites. In addition, the participants valued the social aspects of the collaborative (e.g. meeting up with like-minded individuals), the external driver for change it provided and the legitimacy conferred on it by the Roya College of Surgeons. However, we did not find strong evidence for the level of sharing, partnering and cross-pollination of ideas found to be key mechanisms within some QI collaboratives [17]. This leads us to tentatively suggest that some ‘simpler’ QI problems, such as that addressed in Chole-QuIC, may be just as effectively addressed using lighter-touch QI programme approach with fewer meetings and less emphasis on inter-site collaboration [12]. However, the importance of the social aspects probably precludes a move to a remote-contact only ‘campaign’ approach [32].

At the site level, seen through the lens of NPT, there was certain linearity to the improvement process during Chole-QuIC. Achieving clarity of purpose (coherence) has to be the initial step in any work contingent on the actions of multiple stakeholders, followed by efforts to enrol those stakeholders and legitimise the change (cognitive participation). Colleagues have to be willing to see changes made to practice and seniors have to be willing to allocate time and resources to allow a project leader to drive changes through. Thus, gaining the support of the organisation for the project required that sufficient numbers of key stakeholders viewed the project goals as aligned with their own. In turn, coherence and cognitive participation shape the ability to collectively act to make change happen. Using data to learn from changes made and monitor progress was also required, but ultimately appeared to be the easiest part of the process. This finding diverges somewhat from some recent thought on the challenges of improvement in complex environments [33]. It suggests that sometimes, there may be relatively linear routes to change that are likely to achieve success: it is gaining support for and momentum along these routes that is crucial. In itself, the key change in practice that Chole-QuIC required was comparatively simple: the goal was within the gift of surgeons and managers *if* they all believed it was the right thing to do and *if* contextual pressures did not present issues which took priority over the Chole-QuIC goal. If these conditions were met, other changes would follow relatively easily. This linear process, however, was more easily activated in some organisations than others. It is noteworthy that three of the challenged hospitals were the busiest in terms of surgical volume; conversely, three of the highly successful hospitals had the lowest surgical volume. The size of the challenged organisations, and the presence of multiple surgical teams, meant, first, that achieving coherence across the whole group of surgical stakeholders seemed harder. Second, surgical throughput may be an important constraint on improving practice in an area which, as noted above, relies to a large extent on leads’ ability to make capacity available for extra emergency procedures. The results across the entire cohort (see Table 2) demonstrate high volume centres can achieve significant improvement in care for this patient group, but it should be recognised that challenges in doing so may well be greater for these  hospitals.

Another key point of divergence between highly successful and challenged sites was their willingness and ability to turn ideas into action, and in particular to do this early on in the project. In NPT, the collective action component suggests the importance of both the ease with which new processes can be adopted (interactional workability) and their fit within the local workflow and context (contextual integration). The challenge in quality improvement is to find solutions that are workable and easy to adopt whilst also improving patient care and outcomes. Our findings point to a potentially effective variation on the widely used iterative approach promoted by the Model for Improvement [30]; there needs to be recognition of the time required for generating thoughtful potential solutions, based on an understanding of local systems and context, but this needs to be combined with a subsequent willingness to get on and test these, refining, adapting or discarding as appropriate, in an action-oriented and iterative manner. In our case studies, we found that that the time spent on deliberation upfront made for better solutions that needed fewer rounds of testing, but that openness within the team to testing and iterative adaption was also vital. This aligns with recent thinking in complexity in healthcare organisation, which suggests that the multiplicity of agents involved in any change effort, and the unpredictability of interactions between different parts of a dynamic system, may frustrate even the most thoughtfully developed plans [33]. In such circumstances, acting “scientifically and pragmatically” [34] through a trial-and-error-based approach, of the kind exemplified by the highly successful sites in Chole-QuIC, may be a more effective way of finding a solution that fits local circumstance. Learning from rapid cycles of improvement is a specific skill and provides the foundation for the concept that local QI data holds valuable lessons and supports a ‘turning ideas into action’ mindset. Technical skills in iterative testing and analysing time-series data may be useful, as are the communication skills needed to generate clarity of purpose and motivate colleagues to change. A variety of capabilities are thus required to achieve success in improvement [35]; QI programme designers should be mindful of developing programme-level interventions, such as training and coaching, to support the development of those tasked with leading QI projects at the frontline.

This evaluation has several strengths, including its mixed-methods approach, drawing on a wide range of data to add deeper understanding to the findings of the quantitative evaluation, and the use of a partnered evaluation, capitalising on the rich knowledge of the project team whilst using external oversight to maintain scientific rigour. In particular, the use of disaggregated data demonstrating the differential impact of Chole-QuIC across sites allowed us to examine our qualitative data for systematic differences between the most and least successful sites, such that we could highlight those factors consistently associated with better and worse performance. This enabled us to provide recommendations for others in a field that until now has been characterised by very limited understanding of the ‘active ingredients’ of successful collaboratives, and the work needed to make them work [18]. It also has several limitations. Analysis and interpretation of data took place in the light of the identification of the highly successful and challenged groups, and so was guided by (rather than blind to) the results of the quantitative evaluation. There is also a risk of bias in data collection and analysis by those directly involved in running the collaborative. This was mitigated by partnering with external research expertise, and by deploying a narrow, prospectively defined measure of success to guide analysis. Accordingly, we sought to identify those factors with the most consistent apparent relationship with the impact of Chole-QuIC, whilst also recognising other plausible explanations may exist.

## Conclusion

Collaborative-based quality improvement is a viable strategy for emergency surgery, but its impact rests on the deployment of both effective clinical and improvement strategies by project leads and their colleagues. Achieving clarity of purpose about the proposed changes amongst key stakeholders is a vital precursor to improvement, while protected time and support to enact improvement solutions, and the ability to learn from the experience of doing so were also associated with greater impact within this cohort. We found the use of objective performance data to identify successful sites, and a theoretical lens to interpret the data, helpful in understanding ‘what works’ within surgical quality improvement, and would recommend this as an approach for improvement project evaluations.

## Data Availability

The datasets during and/or analysed during the current study are available from the corresponding author on reasonable request; however, some data will need to be redacted to anonymise all sites and participants.

## Abbreviations

Chole-QuIC: Cholecystectomy Quality Improvement Collaborative
EGS: Emergency General Surgery
IHI: Institute of Healthcare Improvement
NHS: National Health Service
NICE: National Institute of Clinical Excellence
NPT: Normalisation Process Theory
QI: Quality improvement
RCS: Royal College of Surgeons of England
